# Self-care practices and its associated factors among adult diabetes mellitus patients in public hospitals of Sidama region, Southern Ethiopia: a cross-sectional study

**DOI:** 10.11604/pamj.2024.48.36.41041

**Published:** 2024-05-30

**Authors:** Gizachew Ambaw Kassie, Mesay Hailu Dangisso, Dawit Jember Tesfaye

**Affiliations:** 1 Department of Epidemiology and Biostatistics, School of Public Health, College of Medicine and Health Sciences, Wolaita Sodo University, Wolaita Sodo, Ethiopia,; 2 Ethiopian Public Health Institute, Addis Ababa, Ethiopia,; 3 School of Public Health, College of Medicine and Health Science, Hawassa University, Hawassa, Ethiopia

**Keywords:** Diabetes mellitus, self-care practice, Sidama region, Ethiopia

## Abstract

**Introduction:**

poor adherence to diabetes self-care practices can result in adverse health outcomes. Thus, it is important to adapt self-care behaviors to reduce and prevent complications from diabetes mellitus. Therefore, this study aimed to determine the level of diabetes self-care practices and associated factors among adults with diabetes in Ethiopia.

**Methods:**

a health facility-based cross-sectional study was conducted from March to April 2021 in the Sidama region public hospitals. A systematic random sampling technique was used to select 437 diabetic patients. The data were entered using Epi data version 3.1 and analyzed using SPSS version 25. A binary logistic regression analysis was performed, and variables with a p-value <0.05 were considered statistically significant.

**Results:**

in this study, a large number of diabetes patients had inadequate self-care practices. Therefore, it is important to strengthen and establish support systems, such as collaborating with healthcare providers, enlisting the support of family members, and providing health education to improve self-care practices.

**Conclusion:**

this study found that 48.9% of participants had a good level of self-care practice. College graduates and above [AOR: 4.4, 95% CI (1.87, 10.4)], those with strong social support [AOR: 4.6, 95% CI (2.3,10.5)], attendees of health education [AOR: 2.33, 95% CI (1.38,4.6)], those who were on oral hypoglycemic drug [AOR: 0.45, 95% CI (0.24, 0.83)], those who perceived the benefits of self-care [AOR: 0.46, 95% CI (0.25,0.84)], and those who perceived the severity of complications [AOR: 0.56, 95% CI (0.29, 0.77)] were predictors of diabetes self-care practices.

## Introduction

Diabetes mellitus is a significant global health issue, affecting 415 million people worldwide [1]. More than 80% of individuals with diabetes reside in low-income and middle-income nations. This proportion is anticipated to rise to 642 million by 2040, if left unchecked. An estimated 5.0 million fatalities were attributed to diabetes among individuals aged 20-99 years. Of those, over one-third (1.8 million) occurred in individuals under the age of 60 years [1,2]. DM is a significant risk factor for life-threatening conditions like cardiovascular diseases, blindness, kidney failure, and lower-limb amputation [3,4]. Low-income and middle-income countries often have a limited understanding and inadequate prioritization of non-communicable diseases, such as diabetes mellitus. Without effective strategies for managing diabetes and detecting diabetes early in these countries, it is probable that there will be significant increases in complications and prevalence of diabetes [5]. The majority of the serious health problems and premature deaths caused by diabetes are due to both sudden and long-term complications of the disease [3]. It is believed that only 6% of patients with diabetes are free from diabetes-related complications [6]. Diabetes self-care involves a multifaceted approach beyond medication to control blood sugar levels [3]. The World Health Organization (WHO) defines self-care as the efforts carried out by people, families, and communities to improve health prevention, minimize illnesses, and restore health. It includes maintaining a healthy weight, engaging in regular physical activity, regularly checking one's blood sugar, taking care of one's feet, taking medications as prescribed, and avoiding hazards like smoking that might result in the development of diabetic problems [7]. Approximately 95% of patients with diabetes are treated in this manner. Therefore, there should be standardized and efficient procedures for diabetes self-care through personal commitments and through the assistance of healthcare professionals [8,9]. With a total of 2.57 million adult diabetics, Ethiopia is one of the four sub-Saharan African nations with the highest diabetes prevalence rates [10]. Diabetes and its consequences are leading causes of rising patient flow rates and hospitalizations. Despite this, many people with diabetes do not care enough about their condition, which results in poor glucose control and complications [11]. For instance, studies in Ethiopia found that 25.5 percent to 76.8 percent of people practiced self-care [12,13]. This points to the need in the shift of healthcare priorities towards non-communicable diseases as well as the demand for current information on diabetes self-care practices and factors influencing successful diabetes self-care practices [14]. Several of variables, including education level, income, diabetes awareness, participation in the diabetes association, sex, type of treatment, marital status, social support, and age, have been linked to self-care activity adherence [12-17]. Consequently, recognizing risk factors and educating patients about self-care techniques are essential elements of diabetes management to reduce diabetes-related complications and enhance quality of life. Patients with diabetes must comprehend the need to maintain a balanced diet, physical exercise, abstain from cigarettes and alcohol, take their medications as prescribed, maintain good foot cleanliness, and monitored for the development of complications [11]. Self-care practices are crucial for obtaining and sustaining desired blood glucose levels. Studies on diabetic self-care practices have been conducted in Ethiopia. However, the extent of diabetes self-care and risk factors had not been thoroughly investigated in the southern region of Ethiopia. Therefore, this study aimed to evaluate diabetes patients' self-care practices and its predictors among diabetes patients at Sidama region public hospital in southern Ethiopia.

## Methods

**Study setting**: the study was conducted in Sidama regional State public hospitals. Sidama national regional State is one of 11 States in Ethiopia, and its capital is Hawassa City, located 273 km south of Addis Ababa. There are six public hospitals. Each hospital had a diabetes clinic. The regional health bureau is responsible for coordinating the overall healthcare activities of the city.

**Study period**: the study was employed from March to April, 2021.

**Study design**: a facility based cross-sectional study was conducted among diabetes patient in Sidama region public hospitals.

**Study participants**: all diabetic patients who were on diabetic follow-up in Sidama region public hospitals were the source population, and all randomly selected diabetic patients who were on diabetic follow-up visits in Sidama region public hospitals during the study period and who fulfilled the eligibility criteria were the study population.

**Inclusion and exclusion criteria**: all patients with diabetes aged ≥18 years and those with diabetes who had at least two follow-up visits were included in the study. Patients who were critically ill during the study period, those with a documented history of mental illness, those with hearing impairment who were unable to provide the required information by them and pregnant mothers were excluded.

**Sample size determination**: sample was determined using the formula for single population proportion by considering the overall adherence to diabetes self-care practice of 48.1% proportion from the study conducted, in Gondar University referral hospital [15] with 95% level of confidence, 5% margin of error to be tolerated, and 5% non-response rate was added. After adding a 5% non-response rate, the final sample size was 437.

**Sampling technique and procedure**: to select the study unit, a systematic random sampling method with proportional allocation to size (PAS) was used. To achieve this, the sample was distributed proportionally for each hospitals based on the number of patients with diabetes had during the study period. During the study period, 1480 patients with diabetes were on follow-up ate three hospitals. In this study, the sampling interval was calculated by dividing the expected number of diabetics per month by a sample size of 1480/437, which yielded a sampling interval of three until the required sample size was obtained, interviews were conducted every third visit to a follow-up service. At each hospital, the first respondent was selected using lottery.

**Data collection method and procedure**: data were collected using interviewer-administered structured, pretested, validated, and standardized questionnaire. Part I contained sociodemographic characteristics, part II, clinical characteristics of the study subjects, part III, diabetics self-care practices related question, part IV, diabetics health belief-related questions [18], and part V, anthropometric measurements. The revised Summary of Diabetes Self-Care Activities (SDSCA), a standardized and validated tool, was used to measure diabetes self-care activities [9]. This tool consists of dietary practices, physical activity, medication adherence, self-glucose monitoring, and foot care domains.11 items of questions in the domains were used to measure the diabetic self-care activities of the participants. Social Support was measured using the Oslo Social Support Scale (OSSS-3 [19].

**Data quality assurance**: five BSc nurses were recruited to collect the data, and one public health officer supervised and coordinated the process. As soon as the investigators were recruited, they were trained in depth on the data collection instruments, and supervisors supervised the daily activities of data collection.

Operational definition: Participants who scored above the mean value on the 11 self-care practice questions were considered to have good self-care practice. During the past seven days, patients were evaluated on 11 items related to self-care. Each subscale was rated based on seven days, with a higher number representing better self-care operations. In this tool, a score of 0 represents the lowest quality of self-care, whereas a score of 77 indicates the highest quality [15,20].

**Glycemic control**: the level of glycemic control was indicated as controlled when the average fasting blood sugar result is between 70-130 mg/dL and uncontrolled when >130mg/dl or <70mg/dl (i.e. an average of three measures at different visits) [21]. Social support: Was determined as poor support when the sum score of 3-8, as moderate social support when the sum of score 9-11 and as strong support the sum of score? 12 [19].

**Data analysis**: the data were entered into Epi Data version 3.1 and exported to Statistical Packages for Social Sciences (SPSS) version 25.0. Descriptive statistics were used to describe frequency distribution, proportion, measures of central tendency, and dispersion. binary logistic regression was conducted to determine the relationship between each independent variable and diabetic self-care practices, and to select candidate variables for multivariable logistic regression analysis. Variables with a p-value < 0.25 in bi-variable logistic regression analysis were entered into multivariable logistic regression to identify independent predictors of diabetes self-care practices. Multivariable logistic regression was performed to identify the independent predictors of neonatal jaundice. Statistical significance was set at p < 0.05, and the results were presented using text and tables.

**Ethical approval and consent to participate**: the Institutional Review Board (IRB) of Hawassa University College of Medicine and Health Science granted ethical clearance for this study (Ref no; IRB/079/2013). The hospital management committee provided permission and support letters. Participation was consented to anonymously, and written informed consent was obtained from each respondent. To protect confidentiality, participants were assured that their information would not be shared with anyone who was not involved in the study and that their names were not included in the questionnaire.

## Results

**Sociodemographic characteristics of the study participants**: a total of 425 patients with diabetes participated in the study, resulting in a response rate of 97.2%. The median age was 54.6 years with a minimum of 15 years and a maximum of 84 years. Regarding the educational level of the study participants, 131 (30.8%) had attended college education and above. Nearly two-thirds of the study participants, 278 (65.4%) were married, and 324 (76.2%) were urban residents. More than half, 233 (54.9%) of the respondents had reported moderate social support from their families and friends (Table 1).

**Table 1:** socio-demographic characteristics of diabetic patients in Sidama region public hospitals, 2021

Demographic characteristics	Category	Frequency (%)
**Sex**	Male	214(50.4%)
	Female	211(49.6%)
**Age**	15-29	48(11.3%)
	30-44	55(12.9%)
	45-59	216(50.8%)
	>=60	106(24.9%)
**Place of Residence**	Urban	324(76.2%)
	Rural	101(23.8%)
**Current marital status**	Single	48(11.3%)
	Married	278(65.4%)
	Divorced	42(9.9%)
	Widowed	57(13.4%)
**Education status**	No formal education	74(17.4%)
	Primary school	116(27.3%)
	Secondary	104(24.5%)
	College and above	131(30.8%)
**Occupation**	Farmer	37(8.7%)
	Student	24(5.6%)
	Self-employed	99(23.7%)
	Government	139(32.7%)
	Housewife	54(12.7%)
	Daily laborer	25(5.9%)
	0thers	47(11.1%)
**Religion**	Orthodox	136(35.3)
	Muslim	47(9.6%)
	Protestants	193(48%)
	Catholic	49(7.1%)
**Social support**	Good	106 (24.9%)
	Moderate	233 (54.9%)
	Poor	86(20.2%)

**Clinical and behavioral characteristics**: of the total respondents three fourth of the study participants, 321 (75.5%) were type II diabetic patients. The mean (SD) duration of diabetes was 5.44 (3.96) years, with a minimum duration of one year and a maximum duration of 19 years. Approximately 217 (51.1%) patients were taking oral hypoglycemic agents. Majority, 346 (81.4%) had no glucometer at home. Majority, 331 (77.9%) of participants had uncontrolled FBS. Among the respondents, 149(35.1%) had hypertension. Abdominal obesity was observed in 18 (8.4 %) men and 16 (7.5 %) women. Regarding BMI, 114 (26.8%) patients were overweight, and 27 (6.4%) were obese (Table 2).

**Table 2:** clinical and behavioral related characteristics of the diabetics’ patients in Sidama region public hospitals, 2021

Clinical characteristics’	Category	Frequency (%)
**Type of DM**	Type I	104(24.5%)
	Type II	321(75.5%)
**Current treatment**	Insulin	177(41.6%)
	Oral medication	217(51.1%)
	Both	31(7.3%)
**Family history of DM**	Yes	101(23.8%)
	No	298(70.1%)
	I don’t know	26(6.1%)
**Presences of co-morbidity**	Yes	152(35.8%)
	No	273(64.2%)
**Diabetics related complication**	Yes	81(19.2%)
	No	344(80.9%)
**Presence glucometer**	Yes	79(18.6%)
	No	346(81.4%)
**Member of diabetic association**	Yes	93(21.9%)
	No	332(78.1%)
**Attend health education**	Yes	121(28.4%)
	No	304(71.5%)
**History of smoking**	Yes	19 (5.8%)
	No	406 ((94.2%)
**Currently Smoking**	Yes	11(2.6%)
	No	414(97.4%)
**Currently taking alcoholic drink**	Yes	140(32.9%)
	No	285(67.1%)
**Average blood glucose level**	Adequately controlled	94(22.1%)
	Uncontrolled	331(77.9%)
**Knowing current blood glucose level**	Yes	376(88.5%)
	No	49(11.5%)
**Body Mass Index (BMI)**	Normal	246 (57.7%)
	Over weight	114 (26.8%)
	Obese	27 (6.4%)
	Under weight	38 (8.9%)
**Abdominal obesity**	Male	18 (8.4%)
	Female	16 (7.5%)

**Diabetic health beliefs related factors**: more than half of 231 (54.4%) participants reported high perceived susceptibility to diabetes complications, with a mean (SD) score of 15 (±2). The perceived severity of diabetes and its related complications was 208 (48%), with a mean (SD) score of 14.95 (±2.64). Regarding perceived benefits to diabetes self-care practices, 268(63.1%) had high perceived benefits to self-care practices, with a mean score of 15 (±2.25), and 212 (49.9%) had high perceived barriers to diabetes self-care practices.

**Figure 1:**
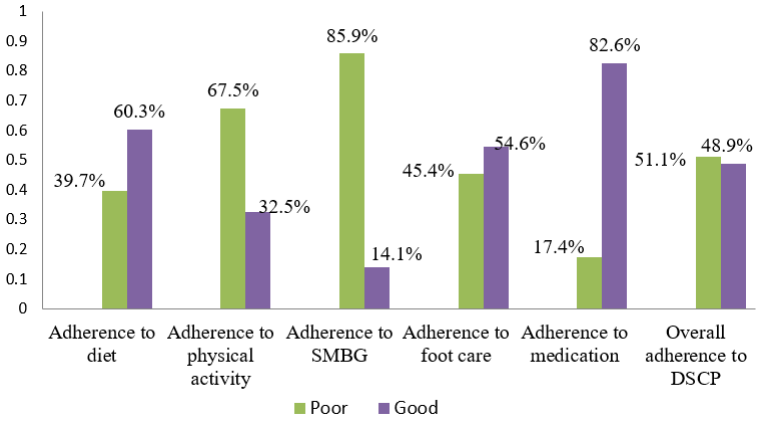
bar graph showing adherence level to components of diabetes self-care practices of diabetic patients on follow-up in Sidama region public hospitals, 2021

**Level of adherence to diabetic self-care practices**: the overall mean (SD) score of diabetic self-care practices among respondents was 3.24 (0.68) days, with a maximum score of seven days and a minimum of zero. Among 425 participants, 208 (48.9%) had good levels of adherence to diabetes self-care practices. Regarding specific domains of diabetic self-care practices, the majority of respondents, and 351 (82.6 %) adhered to antidiabetic medication practices. A total of 262 (60.7%) respondents adhered to recommended diet management. Only 65 (14.1%) participants adhered to the recommended SMBG. Of the total respondents, 138 (32.5%) adhered to physical activity that met the recommended guidelines. Of all study participants, 232 (54.6%) adhered to the recommended diabetic foot care practices (Figure 1).

**Factors contributing to the level of diabetes self-care practice**: in this study, the odds of practicing self-care for diabetes were 4.4 times higher among diabetic patients with an educational status of college and above [AOR: 4.4,95% CI (1.87, 10.4)] than among those who had no formal education. Similarly, study participants who had moderate social support were 2.65 more likely to have good diabetic self-care practices [AOR: 2.65, 95% CI (1.4, 5.1)], and those who had good social support were 4.6 times more likely to have self-care practices [AOR: 4.6, 95% CI (2.3, 10.5)] than diabetic patients who had poor social support. Patients with comorbidities were 2 times more likely to practice diabetic self-care than those without comorbidities [AOR: 2.1, 95% CI (1.12, 3.8)]. In addition, the odds of good diabetic self-care practices were 2.3 times higher among those who attended diabetes education than among those who did not [AOR: 2.33, 95% CI (1.38,4.6)]. Respondents who had a glucometer were two times more likely to engage in good self-care practices than those who did not had a glucometer [AOR: 2.1,95% CI (1.3,4.1)]. Being on oral hypoglycemic agent was 55% less likely [AOR: 0.45, 95% CI (0.24, 0.83)] and being on both oral hypoglycemic agent and insulin were 64% less likely [AOR: 0.36,95% CI (0.13, 0.96)] to have good self-care practices as compared to those patients who take only insulin injections. The odds of good self-care practice were 3.6 times higher among participants who had controlled blood glucose levels compared to those who had uncontrolled blood glucose levels [AOR: 3.6,95% CI (1.9, 6.6)]. Diabetic patients with low perceived severity of diabetes-related complications were 44% less likely to have good diabetic self-care practices [AOR: 0.56, 95% CI (0.29, 0.77)], and those participants who had low perceived benefit were also 54% less likely to practice good self-care than their counterparts (AOR: 0.46, 95% CI (0.25, 0.84)] (Table 3).

**Table 3:** bi-variable and multivariable logistic regression model on factors associated with to DSCP among diabetic’s patients in Sidama region public hospitals, Ethiopia, 2021

Variables	Self-care practice (n=425)			
**Sex**	**Good (%)**	**Poor (%)**	**COR (95%CI)**	**AOR (95%CI)**
**Male**	119 (55.6%)	95 (44.4%)	2(1.36, 2.96) *	1.26(0.75, 2.1)
**Female**	89 (42.2%)	122 (57.8%)	1	1
**Age in years**				
**15-29**	25(52.1%)	23(47.9%)	1.9(0.97, 3.88)	2.4(0.88, 6.45)
**30-44**	31 (56.4%)	24 (43.6%)	2.3(1.19, 4.49) *	1.82(0.77, 1.27)
**45-59**	114(52.8%)	102(47.2%)	2(1.24, 3.23) **	1.8(0.98, 3.27)
**>=60**	38(35.8%)	68(64.2%)	1	1
**Educational Status**				
**No formal education**	19(25.7%)	55(74.3%)	1	1
**Elementary**	39(33.6%)	77(66.4%)	1.4(0.77, 2.9)	1.5(0.66, 3.5)
**High school**	61(58.7%)	43(41.3%)	4.1(2.14, 7.9) **	3.25(1.39, 7.6) *
**Collage & above**	89(67.9%)	42(32.1%)	6.1(3.2, 11.5) **	4.4(1.87, 10.4) **
Current marital status				
**Single**	25 (52.1%)	23 (47.9%)	1	
**Married**	147 (52.9%)	131 (47.1%)	1.3(0.69, 2.23)	1.41(0.61, 3.24)
**Widowed**	22 (38.6%)	35(61.4%)	0.36(0.16, 0.8) *	1.71(0.77, 3.82)
**Divorced**	14 (33.3%)	28(66.7%)	0.53(0.23, 1.24)	0.59(0.25, 1.38)
**Monthly Income ETB**				
**<1000**	20(35.7%)	36(64.3%)	1	
**1000-3000**	91(44.2%)	115(55.8%)	1.42(0.77, 2.6)	0.94(0.42, 2.1)
**>3000**				
**Social support**	97(59.5%)	66(40.5%)	2.7(1.41, 4.9) **	1.42(0.59, 3.4)
**Poor**	28(26.4%)	78(73.6%)	1	1
**Moderate**	126(54%)	107(46%)	3.3(1.98, 5.4)**	2.65(1.4, 5.1)**
**Good**				
**Treatment modality**	54(63.8%)	32(37.2%)	4.7(2.54, 8.7)**	4.6(2.3, 10.8)**
**Insulin**	95(53.7%)	82(53.7%)	1	1
**OHA**	100(46%)	117(54%)	0.74(0.495, 1.1)	0.45(0.24, 0.83)*
**Both**	13(47.9%)	18(58.1%)	0.62(0.29, 1.35)	0.36(0.13,0.96)*
**Member of DM association**	151 (59%)	181 (41%)	1	1
**Yes**	57 (61.3%)	36 (38.7%)	3.9(2.3, 6.7) **	1.02(0.48, 2.18)
**No**				
**Diabetics education**				
**Yes**	87(66.9%)	40(33.1%)	2.82(2, 4.2) **	2.3(1.14, 4.6)*
**No**				
Co-morbidity	121(42%)	183(58%)	1	1
**Yes**	86 (56.6%)	66(43.4%)	1.64(1.1, 2.44) *	2.1(1.12, 3.8)**
**No**	122(45%)	151(55%)	1	1
**Presence of glucometer**				
**Yes**	60(75.9%)	19(24.1%)	4.2(2.4, 7.4) **	2.1(1.1, 4.03) *
**No**	148(43%)	198(57%)	1	1
**Average FBS level**				
**Controlled**	62(66%)	32(34%)	2.5(1.53, 4) **	3.6(1.9, 6.6)**
**Uncontrolled**				
**Susceptibility to DM complication**	146(44%)	185(56%)	1	1
**High**	88 (38.1%)	143 (61.9%)	1	1
**Low**	120 (61.9%)	74 (38.1%)	0.38(0.26, 0.6) *	0.69(0.351, 1.32)
**Perceived Severity**				
**High**	121(58.2%)	87(41.8%)	1	1
**Low**	87(10%)	130(60%)	0.48(0.33, .7) **	0.56(0.29, 0.77)*
**Perceived Benefit**				
**High**	109(64%)	159(36%)	1	1
**Low**	99(35%)	124(65%)	0.8(0.5, 0.9) **	0.46(0.25, 0.84)*

** Statistical significant at p<0.05, * Statistical significance at 0.001

## Discussion

Self-care techniques are crucial for managing blood sugar levels, reducing diabetes related complications, and enhancing quality of life [3]. The level of adherence to diabetic self-care practices was 48.9% (95% CI (44.2, 53.7) in this study, which is consistent with studies done in Kenya [22], Gondar University Hospital [15], Mekelle Ayder referral hospital in 2012 [20], and Benishangul Gumuz region [23] (50.5%, 48.1%, 51.0%, and 54.5%, respectively). However, the finding was higher than the studies done in Dire Dewa (38.3%), Arba Minch (41.2%), Mekelle (37.3%), and Bahirdar (25.5%) [12,16,24,25]. In contrast, it was lower than the adherence levels reported by Dilla (76.8%) [13], Addis Abeba (60.3%) [8] and Nekemitte referral hospital (60.7%) [26]. Variations in the research population's socioeconomic situation, way of life, access to medical services, and educational level may account for these discrepancies.

In this study, adherence to self-care practices was significantly associated with the educational status of patients with diabetes. Patients with DM who were attended college or above had 4.4 times more chances of practicing self-care than those with no formal education. Similar results have been reported from Bangladesh, India, Ghana, Addis Ababa, Mekelle, Gondar, Dire Dawa, and Bahirdar [12,15,16,20,27-30]. Participants with formal education might be able to use their baseline information to practice self-care activities by reading guidelines related to diabetes and implementing professional recommendations [21]. This study found that social support was a strong predictor of adherence to self-care practices. Respondents who received good social support were 4.6 times more likely to engage in good self-care practices compared to those who received poor social support. This finding was consistent with previous studies by Gondar [15], Nekemitte [26], Addis Abeba [8], Bahirdar [12], and Benishangul [23]. A possible explanation for this phenomenon could be that social support from family and friends can help patients deal with problems and provide emotional strength to engage in diabetic self-care practices. Having a glucometer at home was found to be significantly associated with good self-care practices. Respondents who possessed a glucometer were twice as likely to engage in good self-care practices. This finding is consistent with those of studies conducted in Addis Ababa [8], Mekelle [20], and Benishangul [23]. Possibly, having a glucometer at home reinforces regular monitoring of blood glucose levels, which in turn may lead to better awareness of diabetic self-care practices.

Additionally, those who attended diabetes education were two times more likely to practice good self-care than those who did not attend health education from health professionals. This finding is consistent with studies conducted in Dire Dawa [16], Addis Ababa [8], and Benishangul [23], where patients with less frequent information were less likely to engage in good self-care. This may be due to the fact that participants who attend health education are better informed and more likely to follow acceptable standards of self-care activities, such as blood glucose monitoring, diet care, physical activity, and medical care [7]. Patients taking oral medications were 55% less likely to practice good diabetes self-care practices than those taking insulin injections, as per a study conducted in Iran [31], Addis Ababa [28] and Bahirdar [12]. These findings suggest that recipients of insulin injections are more likely to have good self-care practices. This may be because patients receiving insulin injections notice the immediate effects of their treatment and have frequent visits to health facilities. Additionally, patients with diabetes are likely to start taking insulin when their blood sugar level become uncontrolled, which may motivate them to engage in self-care activities to prevent further complications [3,32]. Patients with DM who had co-morbidities were more likely to engage in good diabetes management self-care activities compared to those without any co-morbidity in the current study. This finding is consistent with those of previous studies conducted in Malaysia and Nekemitte, western Ethiopia [26,33]. It is possible that patients with co-morbidities seek more help from their families and are more likely to follow the instructions given by their healthcare providers. Additionally, healthcare providers may prioritize increasing the frequency of follow-up for patients with co-morbidities, which could influence their self-care activities.

Diabetic patients with controlled fasting blood glucose levels were 3.6 times more likely to engage in good diabetes self-care practices than those with uncontrolled blood sugar levels. This may be because good self-care practices help to control and reduce blood glucose levels. Additionally, a patient's knowledge of their blood sugar control status and their attitude towards the health outcomes of adherence to healthy behaviors may influence their actions towards good self-care [34]. Patients with a low perceived severity to diabetic complications and benefits of diabetic self-care practices were less likely to have good diabetic self-care practices. This was consistent with study conducted in Nigeria, which revealed that patients with high perception towards diabetic health beliefs were more likely to engage in good diabetic self-care practices [35]. In addition, it was also consistent with the study report from Dire Dawa [16], Jimma [17], and Mekelle [20], Ethiopia which indicates there was a positive relationship between diabetic health belief and self-care practices. Thus, high perceived severity of DM-related complications and beneficial diabetic self-care practices is helpful for the likelihood of adherence to self-care [35].

**Limitations**: the self-care behaviors of the study participants were dependent on self-reports; as a result there may have been recall and social desirability bias. However, to reduce social desirability bias health professionals recruited as data collectors were not providers of diabetic care at chronic care clinic of each hospital.

## Conclusion

Level of adherence to self-care practices for diabetes in the study area was relatively low. Particularly, self-monitoring of blood glucose and regular exercise was the least practiced diabetes self-care practices. Therefore, it is essential that patients adopt better self-care habits such as monitoring their blood sugar levels, following a healthy and balanced diet, exercising regularly, taking their medication as prescribed and regular check-ups with their healthcare provider. It is equally important that healthcare professionals educate patients on the importance of self-care management. By doing so, we can reduce the number of diabetes-related complications and improve patients' quality of life.

### What is known about this topic

- *Poor adherence to diabetes self-care practices raises the likelihood and prevalence of diabetes related complications, which raises morbidity and death;*
- *Self-care for diabetics is still low in Ethiopia, ranging from 25.5% to 76.8%;*
- *Despite significant advancements in the treatment of diabetes in recent years, many patients still do not enjoy the best results and continue to suffer from devastating complications as a result of poor self-care practices, and there is a paucity of knowledge in the subject area*.

### What this study adds

- *Revealed significant number adults with diabetes had inadequate level of adherence to diabetes self-care;*
- *Identified the factors that influence adherence to diabetes self-care practices that needs attention such as social support, being on oral hypoglycemic, presence of co morbidity, presence of glucometer at home, attending diabetics´ health education, high perception to benefit of diabetics self-care practices and perceived severity to DM related complication were identified as predictors of diabetes self-care practices*.
